# A novel dry-blending method to reduce the coefficient of thermal expansion of polymer templates for OTFT electrodes

**DOI:** 10.3762/bjnano.11.53

**Published:** 2020-04-20

**Authors:** Xiangdong Ye, Bo Tian, Yuxuan Guo, Fan Fan, Anjiang Cai

**Affiliations:** 1 School of Mechanical and Electrical Engineering, Xi’an University of Architecture and Technology, Xi’an 710055, China; 2 Shaanxi Key Laboratory of Nano Materials and Technology, Xi’an 710055, China; 3 School of Automation, Xi'an University of Posts and Telecommunications, Xi’an 710121, China

**Keywords:** coefficient of thermal expansion, dry blending, organic thin-film transistors (OTFTs), OTFT electrodes, PDMS/SiO_2_ composite template

## Abstract

Among the patterning technologies for organic thin-film transistors (OTFTs), the fabrication of OTFT electrodes using polymer templates has attracted much attention. However, deviations in the electrode alignment occur because the coefficient of thermal expansion (CTE) of the polymer template is much higher than the CTE of the dielectric layer. Here, a novel dry-blending method is described in which SiO_2_ nanoparticles are filled into a grooved silicon template, followed by permeation of polydimethylsiloxane (PDMS) into the SiO_2_ nanoparticle gaps. The SiO_2_ nanoparticles in the groove are extracted by curing and peeling off PDMS to prepare a PDMS/SiO_2_ composite template with a nanoparticle content of 83.8 wt %. The composite template has a CTE of 96 ppm/°C, which is a reduction by 69.23% compared with the original PDMS template. Finally, we achieved the alignment of OTFT electrodes using the composite template.

## Introduction

Organic thin-film transistors (OTFTs) provide a platform to construct next-generation large-area, light-weight, flexible, and stretchable optoelectronic applications [1–2], including flexible displays [3], electronic papers [4], sensors [5], and medical applications [6]. Fabricating high-performance OTFTs usually requires that the electrodes on the polymer template are precisely aligned [7]. However, the polymer template has a high coefficient of thermal expansion (CTE), resulting in alignment deviations of the OTFT electrodes [8–9].

Currently, one of the measures to reduce the CTE of polymer templates is wet blending, in which the low-CTE nanomaterial is directly incorporated in a polymer to obtain a composite. Shokrieh et al. [10] carried out a systematic theoretical study to investigate the influence of carbon nanotubes (CNTs) on the CTE of CNT/epoxy, and the results indicate that the addition of 1 wt % CNT causes a significant decrease of the CTE of the matrix. González-Benito et al. [11] used high-energy ball cryomilling to uniformly disperse 5 wt % of titanium dioxide (TiO_2_) nanoparticles with a size of 65 nm within poly(ethylene-*co*-vinyl acetate) (EVA) to subsequently obtain a film of the composite with lower CTE by hot pressing. Ren et al. [12] first prepared a sol–gel precursor by adding tetraethyl orthosilicate (TEOS) to polyvinyl pyrrolidone (PVP), and then synthesized a silica/PVP nanofiber composite by electrospinning. The content of silica nanofibers in the composite is 9.1 wt %, and the CTE was decreased by ca. 40%. Jeyranpour et al. [13] studied the influence of fullerene (C_60_) on the CTE of Araldite LY 5052/Aradur HY 5052 cross-linked epoxy resin by molecular dynamics simulations. The CTE was minimized by adding a maximum of 15.9 wt % fullerene to the LY/HY/C_60_ epoxy system. Liu et al. [14] selected MCM-41 mesoporous silica nanoparticles with a size of 300 nm to be doped into polydimethylsiloxane (PDMS) to prepare a PDMS/MCM-41 nanocomposite. The CTE of the nanocomposite decreased from the initial 301 ppm/°C of PDMS to 241 ppm/°C, when the content of silica in PDMS was increased to 20 wt %. To further reduce the CTE of the polymer template, Kalsoom et al. [15] treated non-porous HPHT microdiamond powder with a size of 2–4 μm with sodium hydroxide and nitric acid followed by intensive washing with deionised water to reduce the tendency to agglomerate. Then, 30 wt % of the synthetic microparticles was added to the acrylate polymer to reduce the CTE of the composite. More recently, Wang et al. [16] hydrolyzed various organic compounds to synthesize a solution of APrTEOS-capped poly(amic acid) (EPI) using the sol–gel method, and then added a maximum content of 32.16 wt % tetramethyl orthosilicate (TMOS) and water (as a diluent) into the EPI to prepare a polyimide–silica hybrid film having a low CTE.

However, because of the poor dispersion of the nanomaterial and the high viscosity of the polymer during wet blending [17–18], the nanomaterial content in the composites is usually low, which causes the CTE of the polymer template to remain high [19]. Hence, we propose a novel dry-blending method, in which the nanomaterial is filled into the grooves of a patterned template first, and then the liquid polymer is poured on the template. As the polymer will permeate into the gaps of the nanomaterial to form the composite, the resultant composite possesses a high content of the nanomaterial. In this paper, a PDMS/SiO_2_ composite template with a SiO_2_ nanoparticle content of 83.8 wt % is prepared via dry blending. Compared to the original PDMS template having a CTE of 312 ppm/°C, the composite template exhibits a CTE of 96 ppm/°C. Using the composite template with the low CTE, we achieved a good alignment of OTFT electrodes.

## Experimental

### Materials

SiO_2_ nanoparticles with a size of 500 nm were provided by XFNANO Materials (Nanjing, China). PDMS (Sylgard 184), consisting of a base and a curing agent, was purchased from Dow Corning Corporation. A silver target of 60 × 5 mm in size and purity of 99.99% as the OTFT electrode material was purchased from ZHNOGNUO New Material Co., Ltd., (Beijing, China). Pentacene as the semiconductor layer was used as purchased from Sigma-Aldrich and dissolved to a concentration of 5% in 1,2-dichlorobenzene (analytical grade). Poly(methyl methacrylate) (PMMA) as the dielectric layer was used as purchased from MicroChem, with a molecular weight of 350,000 and a concentration of 4% in anisole (analytical grade).

### Preparation of the PDMS/SiO_2_ composite template via dry blending

**Figure 1:**
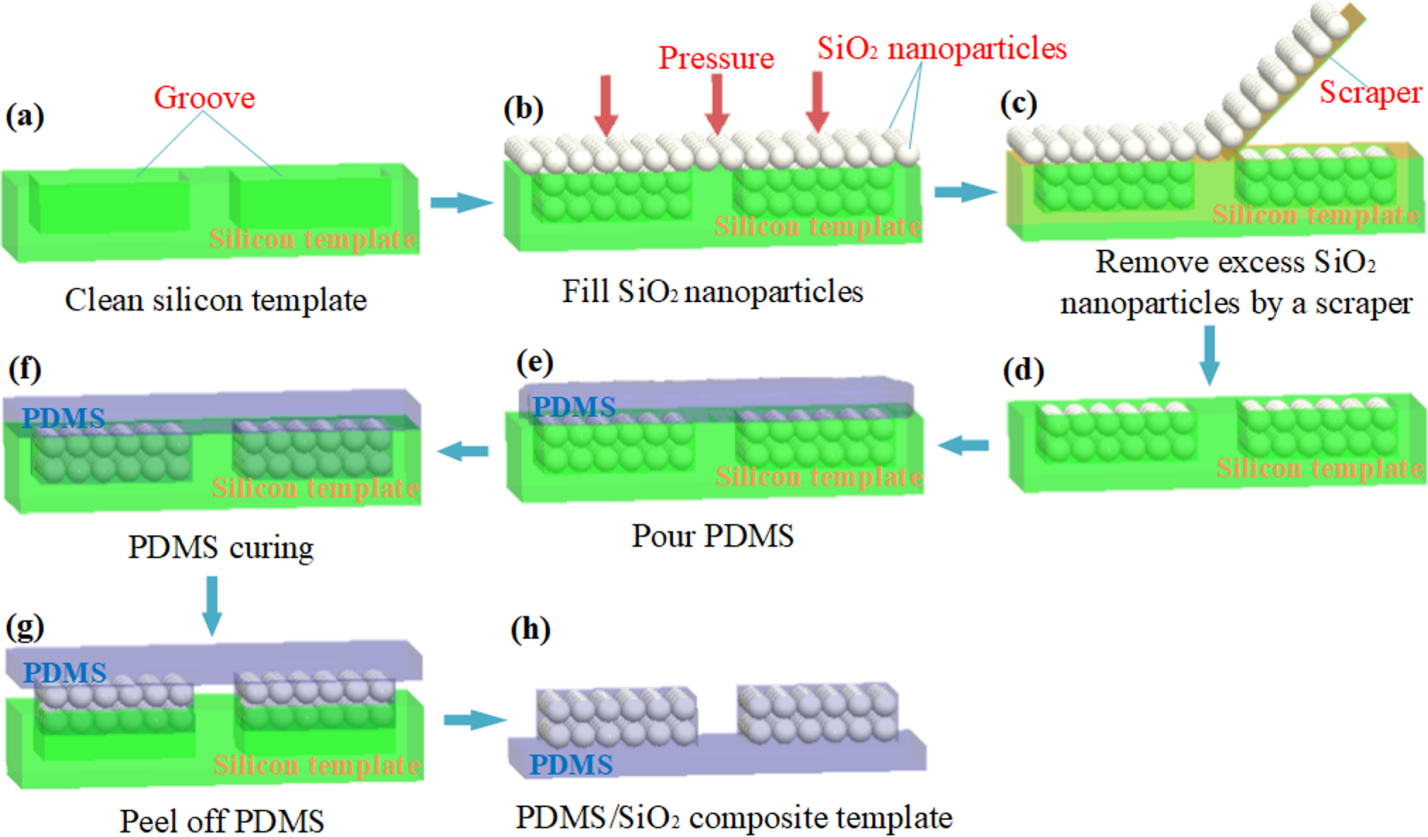
The experimental procedure for preparation of the PDMS/SiO_2_ composite template via dry blending: (a) cleaning of the silicon template; (b) filling with SiO_2_ nanoparticles; (c) removal of excess SiO_2_ nanoparticles; (d) grooves filled with SiO_2_ nanoparticles; (e) covering with PDMS; (f) curing of PDMS; (g) peeling off PDMS; and (h) prepared PDMS/SiO_2_ composite template.

The experimental procedure for preparing the PDMS/SiO_2_ composite template via dry blending is shown in Figure 1.

First, a silicon template prepared by photolithography with a source–drain structure groove was ultrasonically cleaned for 15 min and dried under nitrogen flow. Subsequently, the surface of the silicon template was covered with SiO_2_ nanoparticles and gently pressed with a glass slide to completely fill the SiO_2_ nanoparticles in the groove. The excess SiO_2_ nanoparticles outside the groove of the silicon template were then removed with a scraper. PDMS and the curing agent were then thoroughly mixed at a weight ratio of 10:1 and poured onto the surface of the silicon template. Thereafter, evacuation was performed for 10 min with a vacuum pump, while PDMS penetrated the SiO_2_ nanoparticle gaps. PDMS was then cured at 30 °C for 24 h. Finally, the PDMS/SiO_2_ composite template was prepared by peeling off the PDMS film from the silicon template.

### Characterization

The surface morphology of the PDMS/SiO_2_ composite template and the OTFT electrodes was investigated via scanning electron microscopy (SEM, JSM-6390a, Japan). The thermal expansion of the PDMS/SiO_2_ composite template was examined using a thermomechanical analyzer (TMA, Q400, TA Instruments, New Castle, DE, USA). Five specimens per group with dimensions 20 × 2 × 1 mm were prepared and the average strain was calculated. During the test, the temperature was increased from 20 to 200 °C at a rate of 20 °C/min. The CTE of the PDMS/SiO_2_ composite template was determined based on the curves obtained from the analyzer.

## Results and Discussion

### Surface morphology of the PDMS/SiO_2_ composite template

**Figure 2:**
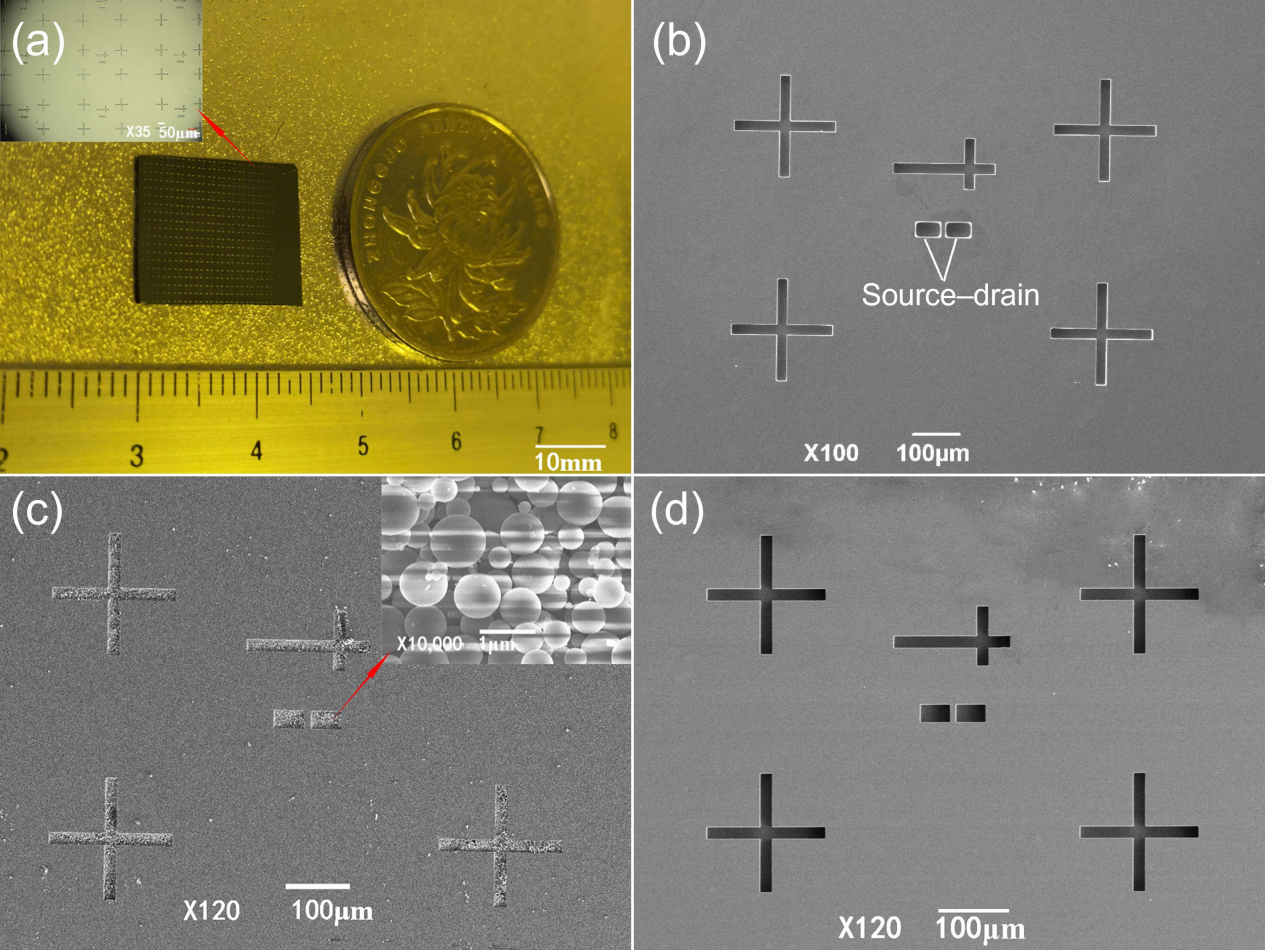
Surface morphology of the silicon template. (a) Physical appearance of the silicon template with source–drain groove. (b) Groove morphology before filling with SiO_2_ nanoparticles. (c) Groove morphology after filling with SiO_2_ nanoparticles and distribution of nanoparticles in the groove. (d) Groove morphology of the silicon template after peeling off the PDMS film.

The prepared silicon template as well as microscopic details before and after filling with SiO_2_ nanoparticles are shown in Figure 2.

**Figure 3:**
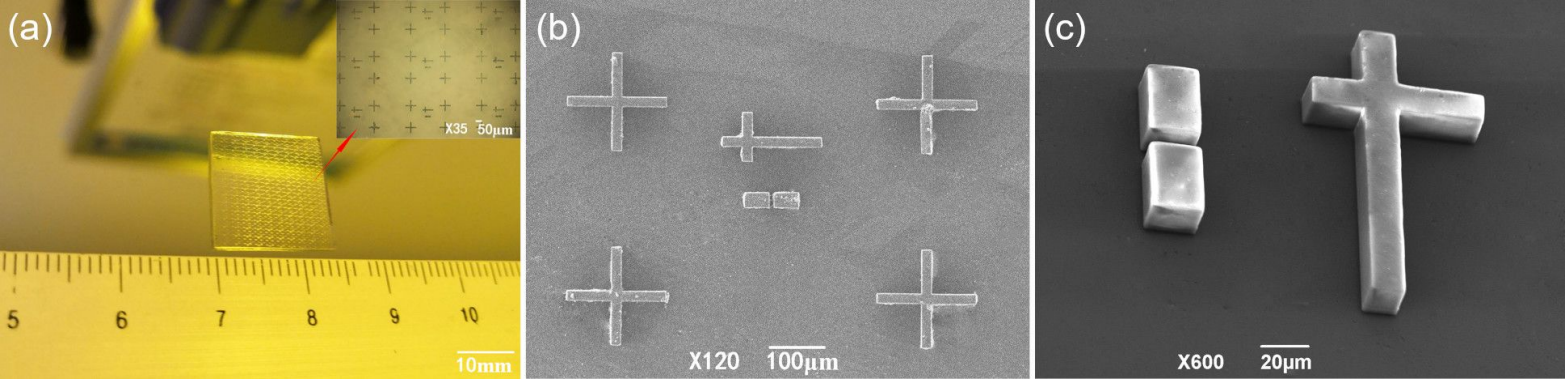
Surface morphology of the PDMS/SiO_2_ composite template. (a) Physical appearance of the PDMS/SiO_2_ composite template. (b) Microstructure of the PDMS/SiO_2_ composite template. (c) Cross-sectional microstructure of the PDMS/SiO_2_ composite template.

Figure 2a shows the physical appearance of the silicon template etched into the source–drain groove structure. The pattern of the array with uniform and regular lines is presented in the upper left corner of Figure 2a. Figure 2b shows the groove morphology before filling with SiO_2_ nanoparticles. It is clear from the groove structure of size 50 × 30 μm that the inside is empty, and the surface of the silicon template is clean. Figure 2c shows the groove morphology after filling with SiO_2_ nanoparticles. Numerous nanoparticles are in the groove, and there is no surplus of nanoparticles on the surface of the silicon template. The magnified image of the distribution of SiO_2_ nanoparticles in the groove at 10000× is shown in the upper right corner of Figure 2c. The nanoparticles filled in the groove are uniformly distributed and regularly arranged. Figure 2d shows the groove morphology of the silicon template after peeling off the PDMS film. There are no nanoparticles in the groove, demonstrating that the PDMS film removes all SiO_2_ nanoparticles from the groove. The PDMS/SiO_2_ composite template prepared via dry blending is shown in Figure 3.

Figure 3a shows the physical appearance of the PDMS/SiO_2_ composite template having a size of 15 × 15 × 1 mm, with the structure of the array on the template surface in the upper right corner. Figure 3b shows the microstructure of the PDMS/SiO_2_ composite template. The width is uniform and the structure is complete, and the surface of the composite template is clean. The cross-sectional morphology of the composite template is shown in Figure 3c. The edges of columnar microstructure are smooth and complete, which indicates that the PDMS template can be peeled off from the silicon template without damaging the structures.

### Calculating the weight fraction of the SiO_2_ nanoparticles

To calculate the weight fraction of the SiO_2_ nanoparticles, it is necessary to first calculate the volume fraction of the SiO_2_ nanoparticles in the groove. As an approximation, we assume that the SiO_2_ nanoparticles are filled with a uniform distribution and regular arrangement according to Figure 1b. SiO_2_ nanoparticles with a particle size of 500 nm are filled into a structure with an etching size of 50 × 30 × 25 μm. Hence, the weight fraction of the SiO_2_ nanoparticles is calculated according to Equation 1 [20]:

[1] $$\omega =\frac{\rho_{\text{m}}\cdot \upsilon }{\rho_{\text{m}}\cdot \upsilon +\rho_{\text{n}}\cdot \left(1-\upsilon \right)}.$$

Here, ω is the weight fraction of the SiO_2_ nanoparticles, ρ_m_ is the density of SiO_2_ (2648 kg·m^−3^ [18]), ρ_n_ is the density of PDMS (965 kg·m^−3^ [20]), and υ is the volume fraction of the SiO_2_ nanoparticles (65.4 vol %). The calculated weight fraction of the SiO_2_ nanoparticles filled via dry blending is 83.8 wt %.

### CTE of the PDMS/SiO_2_ composite template

**Figure 4:**
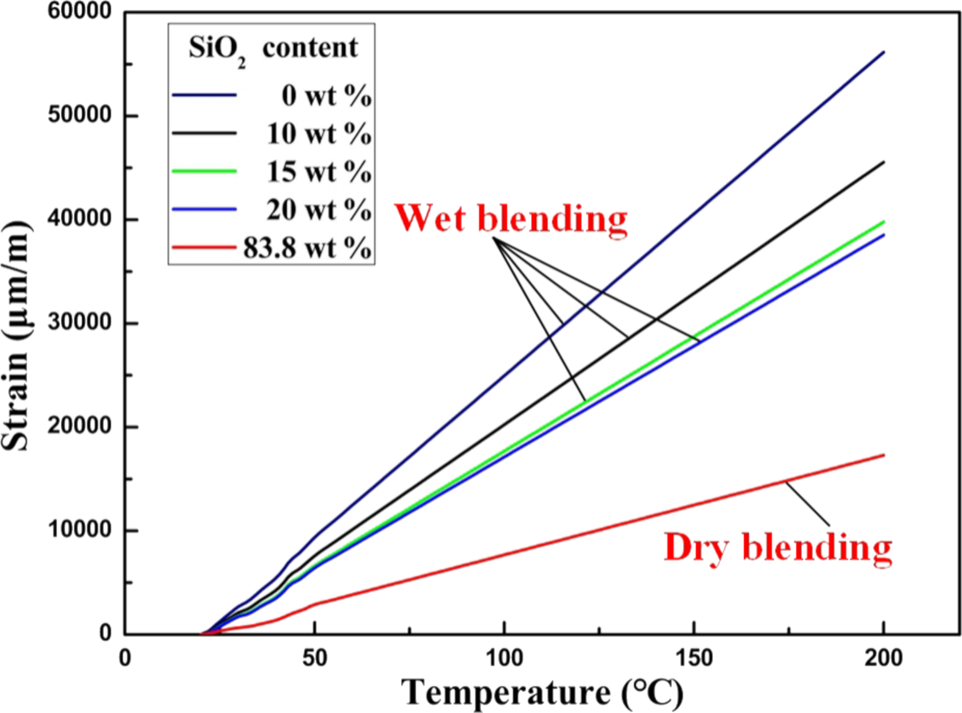
Strain–temperature curves of PDMS/SiO_2_ composite templates prepared via dry blending and wet blending.

The strain–temperature curves of the PDMS/SiO_2_ composite template were investigated with a TMA as shown in Figure 4. For comparison, PDMS/SiO_2_ composite templates with a nanoparticle content of 0 wt %, 10 wt %, 15 wt %, and 20 wt %, respectively, were prepared via wet blending of SiO_2_ nanoparticles and PDMS using ultrasonic technology. Their curves are also shown in Figure 4.

The curves for 0–20 wt % of SiO_2_ nanoparticles in Figure 4 are the strain–temperature curves for the PDMS/SiO_2_ composite template prepared via wet blending, while the curve for 83.8 wt % is the strain–temperature curve for the template prepared via dry blending. The latter curve exhibits the lowest increase in strain with increasing temperature.

**Figure 5:**
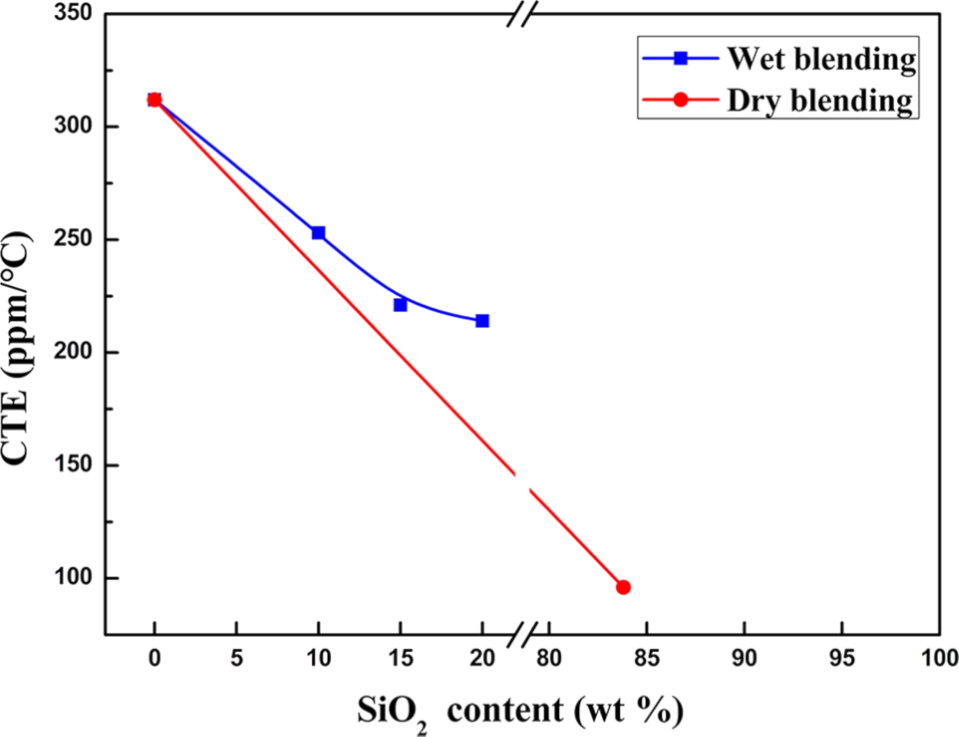
CTE values as a function of the content of SiO_2_ nanoparticles.

Because the temperature increase was not stable in the range from 20 to 50 °C, the curves are distorted. To calculate the CTEs of the PDMS/SiO_2_ composite templates, the slope of the straight lines in the temperature range from 50 to 200 °C was used. The obtained CTE values of the PDMS/SiO_2_ composite templates as a function of the content of the SiO_2_ nanoparticles were calculated and are shown in Figure 5.

The curve for the template prepared via wet blending shows that with a gradual increase in the content of SiO_2_ nanoparticles to 15 wt %, the CTE of the PDMS/SiO_2_ composite template gradually decreases from 312 ppm/°C (PDMS) to 221 ppm/°C. When the content of the SiO_2_ nanoparticles continues to increase to 20 wt %, the CTE of the composite template slowly decreases to 214 ppm/°C. This is primarily because the SiO_2_ nanoparticles in PDMS approach saturation, causing the nanoparticles to be dispersed unevenly, which reduces the influence of the nanoparticles on the CTE. However, the curve for the template prepared via dry blending shows that with increasing content of SiO_2_ nanoparticles, the CTE of the PDMS/SiO_2_ composite template decreases to 96 ppm/°C. This decrease is mainly attributed to two factors. First, the CTE of SiO_2_ is only 0.54 ppm/°C [12]. The higher content of SiO_2_ nanoparticles with a low CTE, the greater the influence on the CTE of PDMS. Second, covalent bonds are formed between SiO_2_ nanoparticles and PDMS and hydrogen bonds are formed between SiO_2_ nanoparticles [14,21]. The higher content of SiO_2_ nanoparticles, the greater the interaction among the bonds between PDMS and SiO_2_ nanoparticles. This restricts the thermal deformation of PDMS. Hence, the CTE of the PDMS/SiO_2_ composite template significantly decreases.

To verify that the CTE of the PDMS/SiO_2_ composite template was reasonable, we compared the CTE with that calculated from a model employing the governing Equation 2 [22]. This equation can be applied to polymer composites filled with one type of nanoparticles:

[2] $$\alpha_{\text{c}}=\alpha_{\text{m}}\left(1-\phi \right)+\alpha_{\text{p}}\phi .$$

Here, α_c_ is the CTE model value of the PDMS/SiO_2_ composite template, α_m_ is the CTE of PDMS (312 ppm/°C), α_p_ is the CTE of SiO_2_ nanoparticles (0.54 ppm/°C), and ϕ is the volume fraction of the SiO_2_ nanoparticles (65.4 vol %). The CTE model value of the composite template was calculated to be 108.3 ppm/°C, which is close to the CTE of the PDMS/SiO_2_ composite template prepared via dry blending.

### Alignment OTFT electrodes

**Figure 6:**
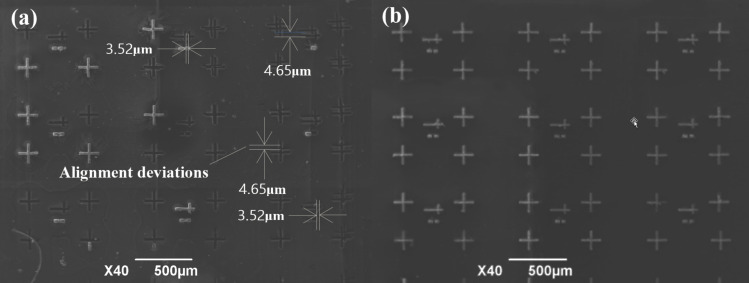
OTFT electrodes alignment. (a) Electrode alignment of the 20 wt % composite template prepared via wet blending. (b) Electrode alignment of the 83.8 wt % composite template prepared via dry blending.

We sputtered an approximately 400 nm thick layer of metallic silver as OTFT electrodes on the surface of the PDMS/SiO_2_ composite template prepared via dry blending. We then used the template with silver electrodes for the alignment of gate and source–drain through a printing process [3,23]. The same experiment was performed using the 20 wt % wet-blended template, as shown in Figure 6.

Figure 6a shows the electrode alignment of the PDMS/SiO_2_ composite template (20 wt %) prepared via wet blending. There are deviations in the alignment of gate and source–drain during the experiment. The vertical deviations are about 4.65 μm and the horizontal deviations are about 3.52 μm. The composite template (83.8 wt %) prepared via dry blending yields a good alignment of gate and source–drain (Figure 6b). The reason for the deviations of the electrode alignment in the wet-blended template is that the CTE of the PDMS/SiO_2_ composite template is 214 ppm/°C, while that of the PMMA dielectric layer to be contacted is 115.2 ppm/°C [24]. The CTE of the PDMS/SiO_2_ composite template prepared via dry blending was 96 ppm/°C, which better matches that of the dielectric layer.

## Conclusion

In this study, we propose a novel dry-blending method in which SiO_2_ nanoparticles are filled into a grooved silicon template, following which PDMS permeates the SiO_2_ nanoparticle gaps. The SiO_2_ nanoparticles in the groove are brought out by curing and peeling off the PDMS to prepare the PDMS/SiO_2_ composite template. The results show that the content of SiO_2_ nanoparticles in the PDMS/SiO_2_ composite template is 83.8 wt %. Moreover, the CTE of the composite template is 96 ppm/°C, and is reduced by 69.23% compared to that of the original PDMS template. Using the dry-blended composite template with the low CTE, alignment between gate and source–drain during the printing process is achieved, which is of great significance in improving the performance of OTFTs. In addition, we believe that dry blending composite templates have potential value in the fabricating process of flexible displays, electronic papers, sensors, and medical applications, and provide new solutions for constructing large-area, light-weight, flexible, and stretchable optoelectronic applications.
